# The appropriate and sequential value of standard radiograph, computed tomography and magnetic resonance imaging to characterize a bone tumor

**DOI:** 10.1038/s41598-022-10218-8

**Published:** 2022-04-13

**Authors:** M. Gaume, S. Chevret, R. Campagna, F. Larousserie, D. Biau

**Affiliations:** 1 Department of Orthopedic Surgery, Cochin Hospital, Université de Paris, AP-HP, Assistance Publique Hôpitaux de Paris, Paris, France; 2 Department of Biostatistics and Clinical Epidemiology, INSERM, Paris University, Paris, France; 3 Department of Radiology, Cochin Hospital, Université de Paris, AP-HP, Assistance Publique Hôpitaux de Paris, Paris, France; 4 Department of Pathology, Cochin Hospital, Université de Paris, AP-HP, Assistance Publique Hôpitaux de Paris, Paris, France

**Keywords:** Cancer, Bone cancer

## Abstract

Radiographs (XR), computed tomography (CT) or magnetic resonance imaging (MRI) are regularly analyzed to determine whether a bone lesion is benign or malignant. An online quiz was created providing 15 cases with a clinical summary, MRI, CT, and XR. After each image, participants were asked to rate the probability (0–100%) the bone tumor was malignant. Order and difficulty of the images were randomly determined. Probability statements regarding the diagnosis were actualized along the sequence of exam, to quantify how the degree of belief changed to account for evidence from those exams. 64 physicians participated and provided 154 assessments from 1 (n = 18) to 3 (n = 44) different cases. After the first image, participants favored the correct malignancy status at 70%; 80% after the second and 80% after the third one. Participants were more likely to favor the correct malignancy status when the lesion was malignant and when first confronted with XR or CT, rather than MRI, though the most predictive factor of correct diagnosis was the difficulty of the case. In conclusion, the additional information provided by successive imaging studies was moderate. XR or CT seemed more appropriate than MRI as first imaging study. Bypassing XR should be discouraged.

## Introduction

Bone tumors are rarely encountered in general practice. Non-neoplastic conditions, metastatic disease, and hematologic malignancies, by far outnumber primary bone tumors^1^. The vast majority of primary bone tumors are benign and malignant primary bone tumors constitute 0.2% of all malignancies in adults. It is essential to distinguish benignity from malignancy of a bone tumor to provide adequate care without undue delays. Imaging plays a key role in navigating the patient through a complex healthcare network^2^.

Standard radiographs (XR), computed tomography (CT) and magnetic resonance imaging (MRI) are the most common imaging modalities that primary care physicians may order. Aggressive behavior is suspected with any of the following characteristics: rapid growth, periosteal reaction, irregular osteolysis, or a soft tissue mass^3^. It is usually thought that the more imaging studies we ask for, the more useful information we have, and the best care we provide. However, significant disadvantages can be opposed: unnecessary costs^4^; undue delays^5^; radiation exposure^6^; unneeded, possibly invasive, follow-up tests; etc. That is why adhering to guidelines for clinical decision making in patients’ candidate to medical imaging procedures are especially important.

Diagnosis of a bone tumor with multiple sequential imaging studies, even when reduced to its simple component “is it benign or malignant?”, is not really binary (ie, true of false): it is a dynamic probabilistic assessment. Ensuing medical decisions, however, such as asking for another imaging modality, referring the patient to a specialized center, or asking for histological confirmation, are binary in essence.

The purpose of this study was to estimate the capability of various imaging modalities and their sequence to differentiate benign from malignant bone tumors; provide the actual diagnosis; and orient the next medical step.

We used probability statements regarding the diagnosis that were actualized as data accumulate, allowing to quantify how the degree of belief could change to account for evidence from those exams.

## Material and methods

### Selection of participants

Participants were selected on a voluntary basis among various oncology groups (EMSOS, GSF), website advertisement (SOFCOT) which is very active to facilitate exchanges between orthopedic surgeons and to reach consensus beneficial to the development of the specialty, and radiology departments.

Potential participants were explained the purpose of the study and the procedure of participation. Informed consent was obtained from all subjects.

The experimental protocol was approved by our ethical institutional committee (CERAPHP Centre). Methods were carried out in accordance with relevant guidelines and regulations.

### Selection of cases

**Figure 1:**
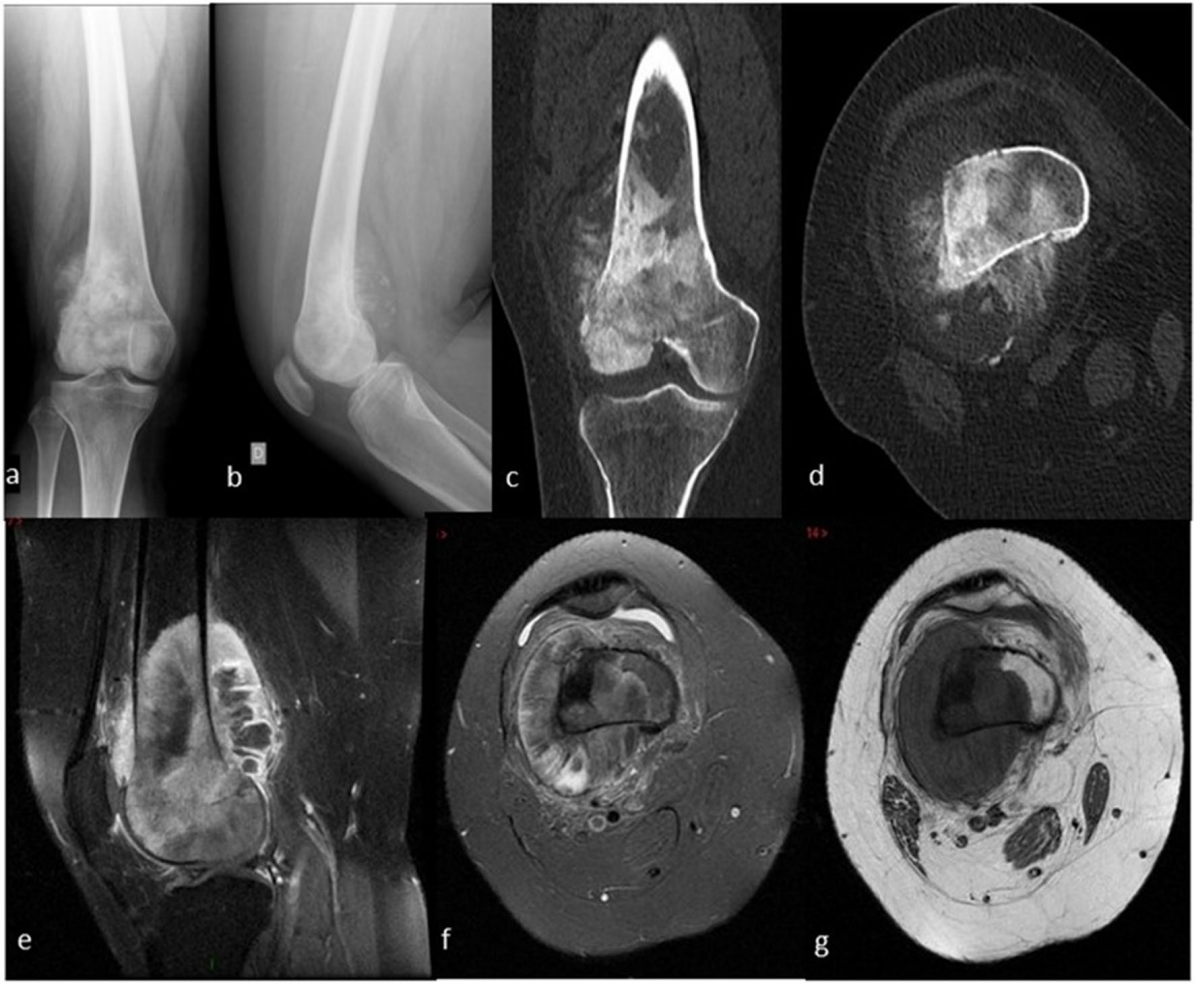
Case of osteosarcoma. A 25 year-old female, no smoker and no drinker, suffered of increasing daily inflammatory right knee pain in the last 6 months. Swelling was noticed few days ago. A tender swelling over the external part of the knee was palpable. Knee mobility was normal. (**1a/b**) Anteroposterior and lateral radiographs of the knee. (**1c/d**) Coronal and axial views on computed tomography. (**1e/g**) T1 sagittal view, T2 fat suppressed axial and T1 sag fat suppressed axial views on magnetic resonance imaging.

**Figure 2:**
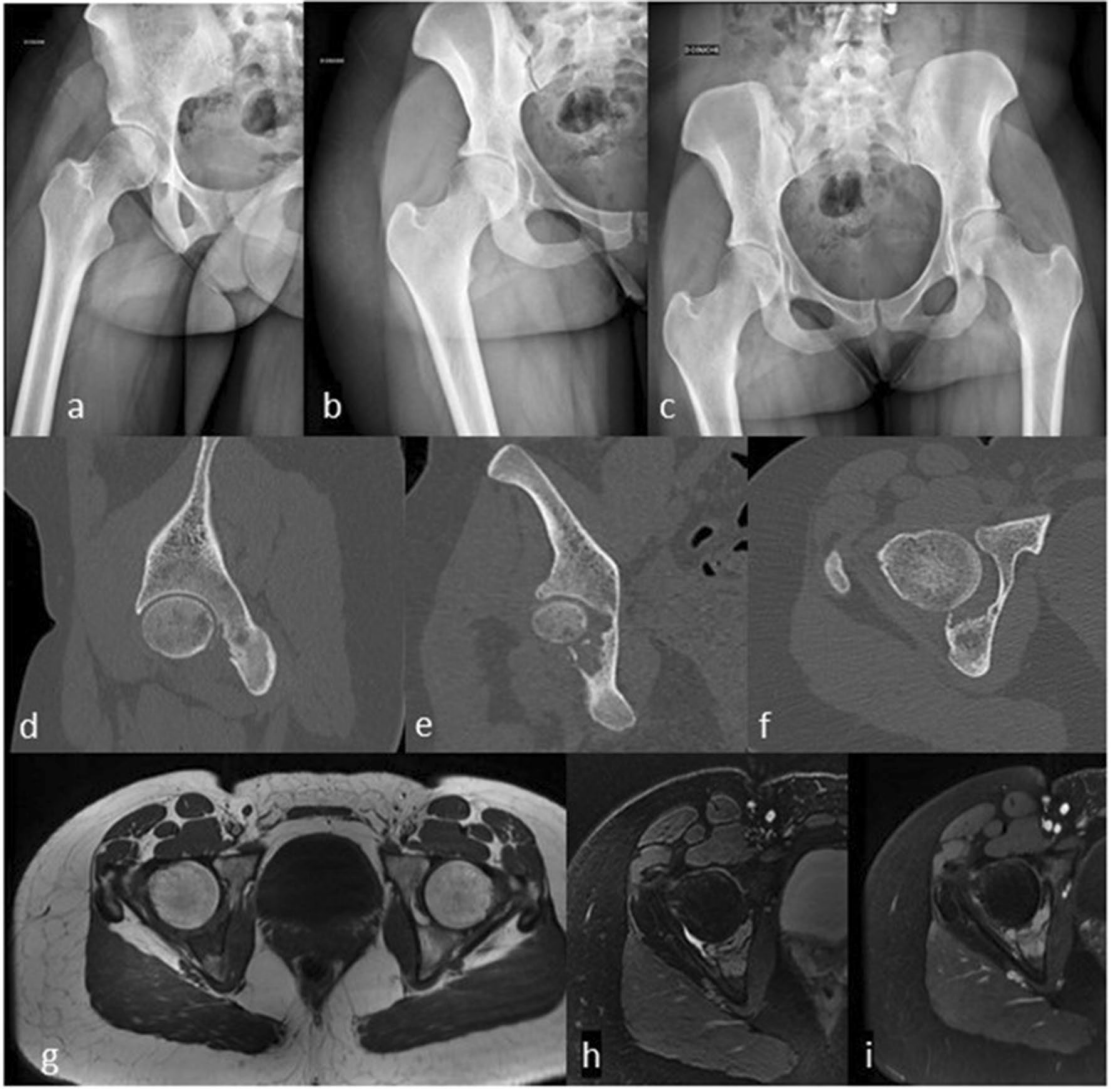
Case of Langerhans cell histiocytosis. A 24 years-old female, no smoker and no drinker has been experiencing left hip pain in the last 6 months relieved by Cortisone injection, with recurrence of the pain 4 months later. No general status loss was observed. There was an inflammatory syndrome on blood test. On palpation, pain was localized on the ischium and on the mobilization of the hip without sensitive or motor dysfunction. (**1a/c**) Standard radiographs of the hip. (**1d/f**) Sagittal, frontal, and axial views on computed tomography. (**1g/i**) T1 and T2 axial and T1gado axial views on magnetic resonance imaging.

One radiologist (RC), one bone tumor pathologist (FL), and one orthopedic oncology surgeon (DB), all with extensive experience in the diagnosis and treatment of bone tumors selected all the cases. Fifteen bone tumor cases (Table 1) were selected among a monocentric bone tumor database. There were no predefined selection criteria but cases were selected to represent common and/or representative bone tumor cases: seven benign and eight malignant bone tumors were chosen». The cases were rated as easy (n = 5), moderate (n = 5), or difficult (n = 5) based on the radiographs, CT and MRI and medical history by the panel of bone tumor experts. For instance, case #2 (Osteosarcoma, Fig. 1) was rated as easy because it is a well know malignant bone tumor, presenting with a typical clinical history; at a common location (distal femur); with typical imaging. On the contrary, case #15 was (Langerhans cell histiocytosis, Fig. 2) was rated as difficult because it is a less known pathology associated with bone tumor, with little relevant clinical information, and poorly informative imaging.

**Table 1:** Estimated probabilities of correct diagnoses along the sequence of exams.

Case_no	Diagnosis	Nature	Level
1	Ewing	Malignant	Difficult
2	Osteosarcoma	Malignant	Easy
3	Chondrosarcoma	Malignant	Moderate
4	Metastasis	Malignant	Difficult
5	Myeloma	Malignant	Easy
6	Lymphoma	Malignant	Moderate
7	Adamantinoma	Malignant	Difficult
8	Chordoma	Malignant	Moderate
9	Giant cell tumour	Benign	Easy
10	Osteoid osteoma	Benign	Difficult
11	Aneurysmal bone cyst	Benign	Moderate
12	Osteochondroma	Benign	Easy
13	Enchondroma	Benign	Moderate
14	Fibrous dysplasia	Benign	Easy
15	Langerhans cell histiocytosis	Benign	Difficult

### Description of the procedure

All readings were done on a designed website. Readers were asked to provide some information regarding their medical curriculum (specialty, date of medical doctorate, years of practice, etc.) and were then given three different cases to read. For each case, they were asked 3 questions. First, they were asked if they thought the tumor was benign or malignant and they were asked to give a probability of confidence to their rating; a “don’t know” answer was allowed. For instance, they could say that the lesion was a malignant bone tumor with 75% probability and a benign bone tumor with a 25% probability. Second, they were asked up to three most likely diagnoses from a drop-down menu list (WHO classification of bone tumors); again, they were asked to associate a probability with each diagnosis; an “other diagnosis” topped up the probabilities to 100%. For instance, they could report that the bone tumor was a giant cell tumor with a 65% probability, an aneurysmal bone cyst with a 30% probability, a telangiectatic osteosarcoma with a 3% probability and something “other” with a remaining 2% probability. Last, they were asked to decide whether they would opt for surveillance/discharge, ask for histological confirmation (open biopsy, needle biopsy, or excisional biopsy), or if they would prefer to have another imaging study before deciding. This only as a matter of making the diagnosis of the bone tumor and not in order to appreciate resecability, extension, or another consideration. For each case, they had to answer those three questions after the first images, the second images, and the third images. The type of images was chosen randomly: it could start with radiographs, CT or MRI; then one of the remaining two imaging studies; then the last one. All images were in JPEG format, with possibility to zoom-in and out.

### Statistical analysis

We considered the percentage of correct answer, as a measure of the physician’s level of evidence on the nature (malignant or not) of the bone tumor, based on actual findings. Given the sequence of imaging, we considered probability trees that represent the probability of correct diagnosis after looking at the first, the second, and the third imaging. We also expressed these probabilities categorically, as very unlikely (less likely than 10%), unlikely (between 10 and 33%), uncertain (between 34 and 66%), likely (between 67 and 90%) or very likely (more likely than 90%), as proposed by Medow and colleagues (Medow 2011). Last, we fitted generalized mixed-effects linear models, to predict the probability of correct diagnosis, adjusting on the severity of the case, the sequence number and the type of imaging, handling the clustering induced by the readers. Mean gain/loss according to specific histologic subtypes were estimated.

All summary statistics reported are medians with interquartile ranges (IQR) or percentages. All analyses were performed on R 3.5.1 (http://www.R-project.org) statistical software packages.

## Results

Sixty-four participants (60%) accepted to take the survey over a total of 106 individuals who were invited by e-mail. Overall, 64 physicians, 44 (69%) seniors and 20 (31%) juniors (i.e., less than 3 years of independent decision making), participated to the study. There were 44 (69%) orthopedic surgeons, 17 (27%) radiologists, two (3%) pathologists, and one other. Fifty-seven (89%) worked in a teaching hospital, 33 at a cancer center (52%); 31 (48%) had sarcoma patients under their care. They provided a total of 154 assessments from 1 (n = 18), 2 (n = 2) or 3 (n = 44) cases each.

**Figure 3:**
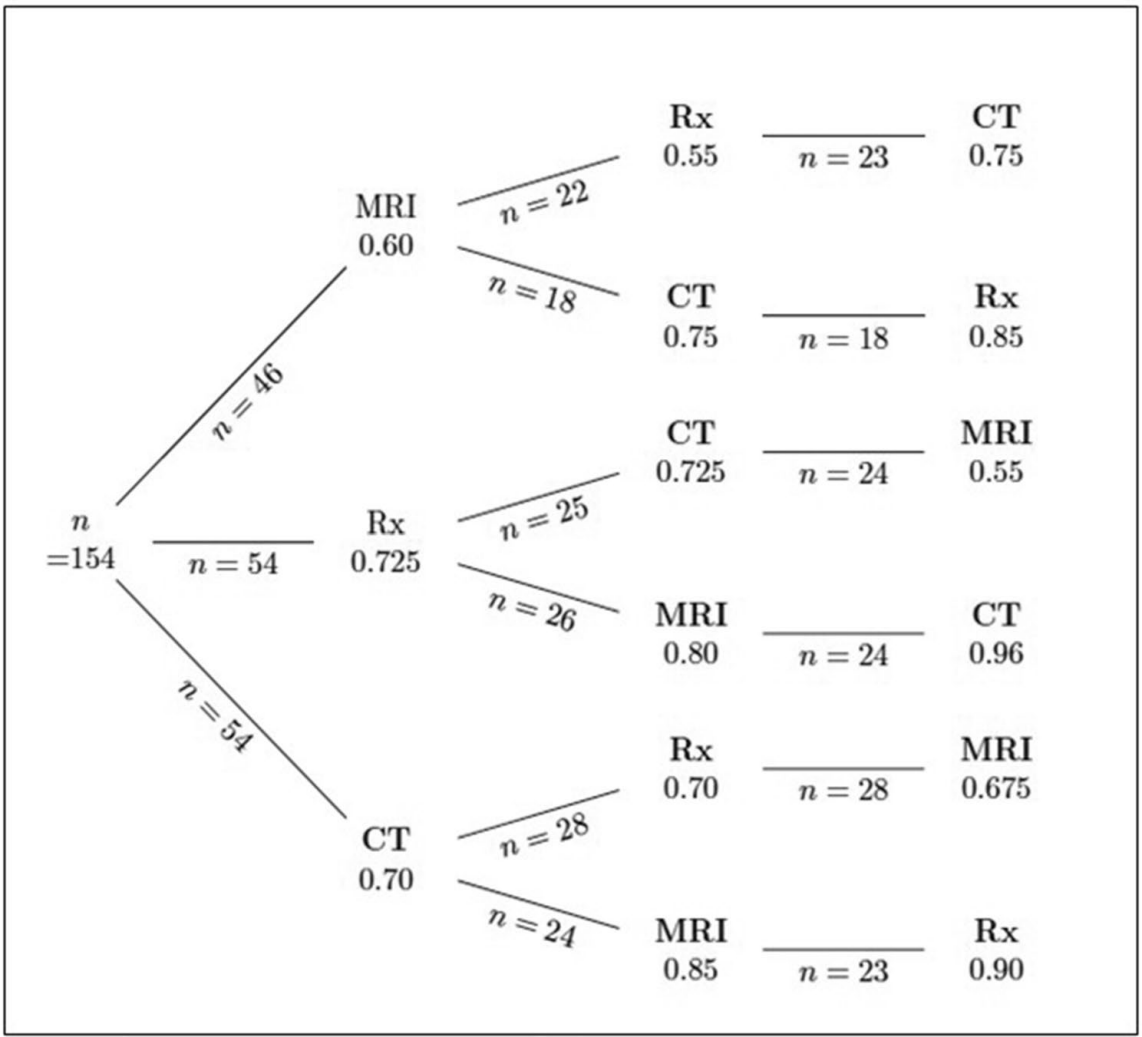
Probabilities of correct diagnosis along the sequence of exams.

After the first imaging study, participants favored the correct malignancy status at 70% (IQR: 27.5–90) (Table 2); after the second imaging study, participants favored the correct malignancy status at 80% (IQR: 40–95); after the third and last imaging study, participants favored the correct malignancy status at 80% (IQR: 40–99) (Table 3). Participants were more likely to favor the correct malignancy status when facing a malignant lesion than when it was a benign lesion (Table 4), though the most predictive factor of correct diagnosis was the difficulty of the case (*p* = 0.0047). The reading sequence also affected the probability of readers to find the correct malignancy status (Fig. 3). Starting with a CT or a XR allowed a better appreciation of malignancy status. The type of imaging studies was associated with improved diagnosis for CT (estimate = + 1.07, SE = 3.81) and MRI with decreased probability of correct diagnosis for MRI (estimate = − 0.37, SE = 3.8).

**Table 2:** Description of percentages of correct diagnoses over time according to the first imaging.

	CT n = 54	MRI n = 46	XR n = 54
First exam	N = 51	N = 42	N = 50
Pr correct diagnosis	0.7 [0.27–0.90]	0.6 [0.25–0.90]	0.72 [0.35–0.90]
Very unlikely	4 (9%)	2 (5%)	8 (16%)
Unlikely	12 (24%)	12 (29%)	5 (10%)
Uncertain	8 (16%)	9 (21%)	10 (20%)
Likely	16 (31%)	13 (31%)	15 (30%)
Very likely	11 (22%)	6 (14%)	12 (24%)

Second exam	N = 52	n = 40	n = 51
Pr correct diagnosis	0.8 [0.47–0.9]	0.80 [0.23–0.91]	0.85 [0.5–0.85]
Very unlikely	6 (12%)	5 (12%)	4 (8%)
Unlikely	6 (12%)	8(20%)	4 (8%)
Uncertain	6 (12%)	5 (13%)	11(22%)
Likely	22 (42%)	12 (30%)	15 (29%)
Very likely	12 (23%)	10 (25%)	17 (33%)

Third exam	N = 51	N = 41	N = 48
Pr correct diagnosis	0.8 [0.4–0.95]	0.8 [0.3–0.95]	0.85 [0.43–1]
Very unlikely	7 (14%)	3 (7%)	5 (10%)
Unlikely	5 (10%)	8 (20%)	6 (13%)
Uncertain	10 (20%)	5 (12%)	9 (19%)
Likely	14 (27%)	13 (32%)	7 (15%)
Very likely	15 (30%)	12 (30%)	21(44%)

*CT* Computed tomography, *XR* Standard radiograph, *MRI* Magnetic resonance imaging.

**Table 3:** Estimated probabilities of correct diagnoses along the sequence of exams.

	Number of measures	Median [IQR]
**First exam**		
Very unlikely	14 (10%)	
Unlikely	29 (20%)	
Uncertain	27 (19%)	
Likely	44 (31%)	
Very likely	29 (20%)	
**Second exam**		
Pr correct diagnosis	143	0.80 [0.40–0.95]
Very unlikely	15 (10%)	
Unlikely	18 (12%)	
Uncertain	22 (15%)	
Likely	49 (34%)	
Very likely	39 (27%)	
**Third exam**		
Pr correct diagnosis	140	0.80 [0.40–0.99]
Very unlikely	15 (11%)	
Unlikely	19 (14%)	
Uncertain	24 (17%)	
Likely	34 (24%)	
Very likely	48 (34%)

**Table 4:** Estimated probabilities of correct diagnoses along the sequence of exams according to the underlying diagnosis.

	Benign, n = 67	Malignant, n = 87	
First exam	n = 58	n = 85	
Pr correct diagnosis	0.55 [0.30–0.90]	0.75 [0.25–0.90]	0.52
Very unlikely	4 (7%)	10 (12%)	
Unlikely	12 (21%)	17 (20%)	
Uncertain	16 (28%)	11 (13%)	
Likely	16 (28%)	28 (33%)	
Very likely	10 (17%)	19 (22%)	

Second exam	n = 60	n = 83	
Pr correct diagnosis	0.65 [0.40–0.9125]	0.80 [0.50–0.95]	0.14
Very unlikely	6 (10%)	9 (11%)	
Unlikely	7 (12%)	11(13%)	
Uncertain	17 (28%)	5 (6%)	
Likely	15 (25%)	34 (41%)	
Very likely	15 (25%)	24 (29%)	

Third exam	n = 62	n = 78	
Pr correct diagnosis	0.775 [0.425–0.9725]	0.85 [0.40–0.99]	0.61
Very unlikely	7 (11%)	8 (10%)	
Unlikely	8 (13%)	11 (14%)	
Uncertain	11 (18%)	13 (17%)	
Likely	15 (24%)	19 (24%)	
Very likely	21 (34%)	27 (35%)

When considering all the parameters jointly in a multivariable model (Table 5), the most predictive factor of correct diagnosis was the difficulty of the case (with a mean difference in correct probability that decreased by − 18% when the case was moderately difficult, and by − 23% when it was difficult, *p* < 0.0001); by contrast, the probability of correct diagnoses was poorly affected by the malignancy of the case (by 3.2%, *p* = 0.32) or by the specialty of the physician (with a 1.6% improvement for radiologists, *p* = 0.85, and of 0.4% for orthopedists, *p* = 0.96).

**Table 5:** Univariable and multivariable mixed regression models for the probability of correct diagnoses.

	Univariable		Multivariable	
	Estimate (SE)	*p* value	Estimate (SE)	*p* value
**Imaging modality**				
XR	Ref	–	Ref	–
CT	0.58 (3.68)	0.87	1.06 (3.82)	0.78
MRI	− 0.51 (3.66)	0.89	− 0.72 (3.80)	0.85
**Sequence**				
First reading	Ref		Ref	
Second reading	6.34 (3.63)	0.08	6.54 (3.79)	0.08
Third reading	6.17 (3.66)	0.09	6.29 (3.82)	0.10
Malignancy (malignant)	3.94 (3.3)	0.23	3.17 (3.17)	0.32
**Difficulty**				
Easy	Ref	–	ref	–
Moderate	− 17.9 (3.8)	< 0.0001	− 18.08 (3.89)	< 0.0001
Difficult	− 24.7 (4.1)	< 0.0001	− 22.77 (4.01)	< 0.0001
Experience (senior )	3.47 (5)	0.48	4.90 (3.44)	0.15
**Physician specialty**				
Radiology	5.35 (11.97)	0.65	1.56 (8.33)	0.85
Orthopaedics	7.86 (11.39)	0.49	0.38 (7.91)	0.96
Other	Ref		Ref	

*All univariable models were adjusted for reader as a random variable.

When considering only the easy cases, the main factors associated with correct diagnoses were the sequence number of reading with a mean gain of 13% for the second (*p* = 0.02), and + 11% for the third readings (*p* = 0.04), and the experience of the reader (mean gain of 13% if senior, *p* = 0.008); no effect of the reader’s specialty was observed (*p* = 0.49). By contrast, in malignant cases, correct diagnoses, still decreased in case of difficult (*p* < 0.0001) or moderate (*p* = 0.0014) cases, were also decreased in orthopedists (*p* = 0.020) and radiologists (*p* = 0.027) by − 27% each.

In such malignancies, correct diagnosis was increased in case of osteosarcoma (with a mean gain in correct probability of 44.8% *p* < 0.0001), chordoma (mean gain of 42.7%), and metastasis (mean gain of 40.5%, *p* < 0.0001) while it was decreased in case of chondrosarcoma (by − 10.6%), though not significantly (*p* = 0.10). In benign tumors, the probability of correct diagnosis increased in case of chondroma (+ 36.2%, *p* < 0.0001), fibrous dysplasia (+ 35.9%, *p* < 0.0001), giant cell tumor (+ 32.9%, *p* < 0.0001) while it decreased in case of Langerhans cell histiocytosis (− 12.6% *p* = 0.048).

When confronted with a malignant tumor, the probability to ask for histological confirmation increased from 38% after the first imaging series, then 62% after the second imaging series, to 80% after the third and last imaging series.

After the first image, the mostly request concerned imaging, with 105 (68%) requests, while 44 (29%) concerned histopathological findings and 5 (3%) suggested surveillance. Such demands heavily depended on the underlying nature of the case (*p* = 0.003), with, in case of malignancies, 38% of requests for histopathological findings, 61% for imaging and 1% for surveillance versus 16%, 78% and 6%, respectively.

After the second image, the most asked type of additional exam switched to the histopathology (54%), followed by imaging (38%), then surveillance (9%); such results were still affected by the type of underlying tumor with 62% histology, 34% imaging and 4% surveillance in case of malignancy versus 43%, 42% and 15% otherwise, respectively.

Last, after the third image, requested histopathological findings increased up to 69% (101/147), while surveillance reached 19% (n = 28) of requests and imaging decreased down to 12% (n = 18). Once again, those findings depended on the underlying malignancy, with 80% of requests for histology, 12% imaging and 7% surveillance in case of malignancy, versus 54%, 12% and 33% otherwise.

## Discussion

We were interested in evaluating how the diagnosis of bone tumors is actualized according to the knowledge provided by new imaging. The answers to our Quiz confirmed that XR was the most effective tool in primary evaluation. The additional information of CT and MRI provided moderate benefits to confirm the malignancy of the lesion. Thus, a patient with a suspected malignant lesion on the XR should be referred without delay to a reference center. CT and MRI are not necessary before then. Conversely, a CT or an MRI were useful to confirm a suspected benign bone tumor. These findings should help to reduce unnecessary cost and undue delays.

These results are borne out by Nystrom et al.^7^. In their prospective series, 32.4% of all advanced imaging prior to referral were inappropriate, including 36.5% CT and 26.7% MRI. The most common reason for inappropriate MRI was imaging of a bone tumor that possessed clearly benign characteristics on radiography and/or CT. 30% of MRI and 24% of CT were ordered without prior radiographic evaluation.

Recently, Ribeiro et al. proposed an evidence-based systematic approach to solitary bone tumor characterization based on demographic, clinical, and imaging features. Three features (Lodwick-Madewell grade III, aggressive periosteal reaction, and suspicion of metastatic disease) had a mean frequency of associated malignancy over 80% (72/88). Thus, lesions presenting any of these signs should be considered malignant until proven otherwise. These conclusions are consistent with our results^8^.

Patient’s medical history and findings on physical examination usually contributes limited information^9^. If malignancy is suspected, age is a relevant epidemiological factor in determining the precise diagnosis. For example, Ewing's tumor is frequent before the age of 20 years, and osteosarcoma in the second decade. A destructive lesion of the bone in patient over 40 years old is likely to indicate metastasis to bone or myeloma. The presence of altered function is also relevant for malignancy. The presence of a soft-tissue mass should make the physician suspect an aggressive bone tumor with an extra osseous extension.

In this context, imaging play a major role in the initial diagnostic of bone tumor. In February 2018, the work group of Musculoskeletal Tumor Society and American Academy of Orthopedic recommended plain radiographs as the initial evaluation for a possible bone tumor^10^.

Based on our Quiz, XR are the most effective tool in primary evaluation with 72.5% [IQR, 35–90] of correct risk of malignancy status at first imaging status. This result is consistent with other studies^11,12^. Onal et al.^13^ assessed that conventional imaging criteria for bone malignancy can be misleading when applied to MRI or CT. When the periosteal reaction was considered, XR was the best modality to detect the malignant type of periosteal reaction. MRI and CT were misleading; either by not detecting or undergrading periosteal reaction. The Modified Lodwick-Madewell Grading System^14^ based on XR is correlated with risk of malignancy from grade I benign) to grade II (moderate risk of malignancy) and grade II (high likelihood of malignancy).

Indistinct margins are usually typical of malignant lesions but may be seen in aggressive benign bone tumors (giant cell tumors for instance); well defined margins may, although rarely, be seen in low-grade malignant bone lesions also (chondrosarcoma for instance).

The radiographic margins of bone tumor is an important indicator to whether it is benign or malignant^15^. The periosteal reaction carries a high correlation with malignancy. A disrupted periosteal proliferation can be seen in bone sarcomas, especially if laminated (“lamellated periostal reaction”) and speculated (“periostal spiculations”). Extensive cortical breakthrough suggests malignancy, especially when tumor extension into the adjacent soft tissues is demonstrated on the XR; a soft tissue mass, however, is more likely to be appreciated on MRI. Tumor size has also to be taken in consideration. In general, the larger the lesion, the more likely it is malignant. This imaging has also the advantage of being quick, noninvasive and low cost. On the contrary, malignant bone tumors are always well defined on MRI which could lead to inappropriate reassurance.

CT provide useful complementary information in conjunction with radiographs. After a second imaging study with CT, participants reached the correct malignancy status in 10% more. CT is the best method for assessing matrix mineralization (osteoid or chondroid), and periosteal reaction^16,17^. It is particularly helpful in evaluating bone tumors in complex anatomical sites, such as the spine, pelvis and hindfoot^18^. In Wurtz et al. study^19^, 27% of primary bone sarcoma of the pelvis had an initial misinterpreted XR by general practitioners, radiologists, and orthopedic surgeons.

The results of this Quiz underline that MRI is not appropriate at first imaging study and does not contribute to improve the rate of correct malignant diagnosis. MRI facilitates evaluation of intraosseous extension, matrix, soft tissues and vascular involvement. MRI is also particularly useful in the detection of skip lesions. Thus, the great potential of MRI is in the staging of tumors, allowing the surgeon to operate with greater confidence. Aboulafia et al.^4^ reported that MRI were obtained in 76% of patients before referral. It was the case in 71% of patients in Miller series^20^, with 17% found to be wasteful. Even if the indications were sound, technical inadequacy often required to repeat the study (failure to image the correct anatomic site or the entire compartment containing a suspected tumor, or images that lack intravenous contrast to properly evaluate the tumor). In Martin’s study^21^, inappropriate MRI use was not as widespread probably thanks to practice habits and education.

In the future, the advent of probabilistic classifier based on naïve Bayes machin Bayesian model that incorporates radiographic observations and demographic characteristics to rank bone tumor diagnoses may help to improve the characterization of bone tumor^22^.

One of the weaknesses of this study was the low number of participants (64) but it was difficult to recruit more qualified physicians interested in the bone tumor field. Moreover, the bone tumors were heterogenous (15 cases) and it made it difficult to generalize the conclusions to all lesions of the same histological type. Finally, one of the limitations was the site of the bone tumor exclusively focused on limbs or pelvis. Cases of spinal locations might have an influence on the study results.

## Conclusion

Despite the wide variety of imaging modalities available, XR should be ordered first to assess malignancy status of a bone tumor. Patients with suspected malignant lesion should be referred to an oncology referral center based on their XR only to avoid management delay. The additional diagnostic information provided by advanced imaging studies, CT and MRI, is moderate and should not delay the medical care. The relevance of further imaging studies should be determined at a referral center before a biopsy is performed. This study should help clinicians to provide better care of patients in case of bone tumor suspicion.

## Abbreviations

XR: Standard radiograph
CT: Computed tomography
MRI: Magnetic resonance imaging
EMSOS: European Musculo-Skeletal Oncology Society
GSF: Groupe Sarcome Français
SOFCOT: Société Française de Chirurgie Orthopédique et Traumatologie
WHO: World Heath Society
IQR: Interquartile range
